# Supplementary material for: mRNA poly(A)-tail length is a battleground for coronavirus–host competition
Source: bioRxiv. 2025 Oct 26:2025.10.09.680815. Preprint. [Version 2] doi: 10.1101/2025.10.09.680815 (PMC12632535; doi:10.1101/2025.10.09.680815)
Supplement: Supplement 1 [file NIHPP2025.10.09.680815v2-supplement-1.pdf]

bioRxiv preprint doi: https://doi.org/10.1101/2025.10.09.680815; this version posted October 26, 2025. The copyright holder for this preprint (which was not certified by peer review) is the author/funder, who has granted bioRxiv a license to display the preprint in perpetuity. It is made available under aCC-BY 4.0 International license.

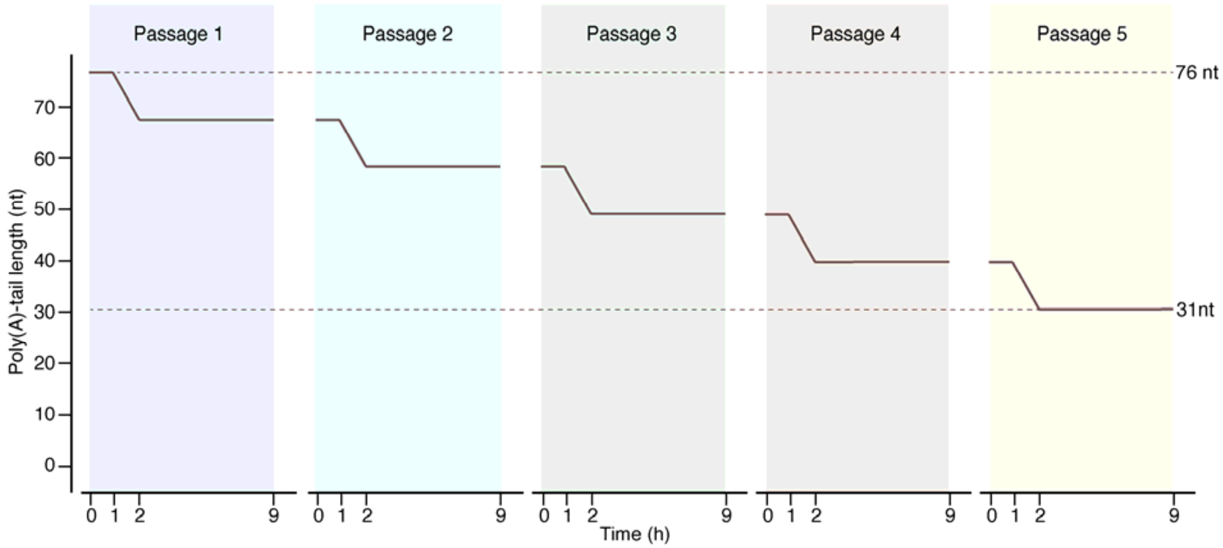

Figure S1. Poly(A)-tail length of viral mRNAs is expected to decrease during successive infections

A best-case estimate of poly(A)-tail length dynamics in viral mRNAs. Plotted are predicted poly(A)-tail length changes of viral mRNAs under optimal settings. During the first hour post-infection, viral mRNAs enter the cytoplasm, and their poly(A) tails remain protected within the capsid. In the second hour, as the viral genomic mRNA undergoes translation, the poly(A) tail becomes susceptible to deadenylation. In the most favorable scenario, assuming one of the slowest deadenylation rates reported for TOP mRNAs[4] (0.15 nt/min), the tail would shorten by ~9 nt. In the subsequent hours, once the viral RdRp is synthesized, the poly(A) tail is effectively stabilized as a poly(U) tract, and efficient encapsidation of the genomic mRNA into new virions prevents further deadenylation. Under this scheme, a genomic mRNA that begins with a 76-nt poly(A)-tail length is projected to have a 31-nt poly(A)-tail length by passage 5.

bioRxiv preprint doi: https://doi.org/10.1101/2025.10.09.680815; this version posted October 26, 2025. The copyright holder for this preprint (which was not certified by peer review) is the author/funder, who has granted bioRxiv a license to display the preprint in perpetuity. It is made available under aCC-BY 4.0 International license.

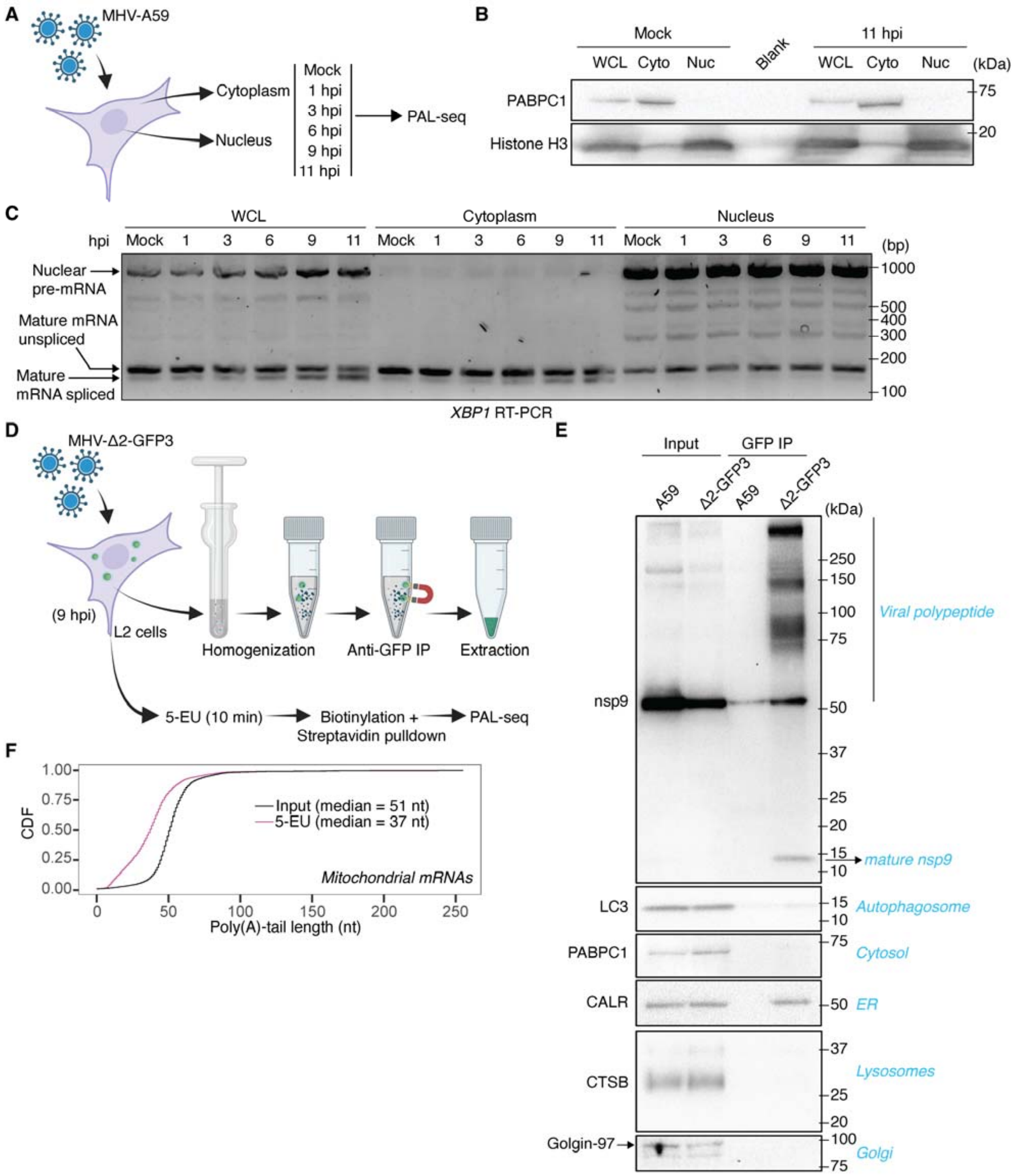

Figure S2. Poly(A)-tail extension of viral mRNAs occurs in double membrane vesicles

(A) Outline of infection time course and fractionation.

(B) Assessment of subcellular fractionation. Shown are immunoblots of whole-cell lysates (WCL), cytoplasmic lysates (Cyto), and nuclear lysates (Nuc) of mock and infected cells (11 hpi) probed for a cytoplasmic marker (PABPC1) as well as a nuclear marker (Histone H3).

(C) Assessment of subcellular fractionation by processing of Xbp1 mRNA. Shown is an agarose gel of RT-PCR products for Xbp1 mRNA in WCLs, cytoplasmic lysates, and nuclear lysates of mock or MHV-infected cells.

bioRxiv preprint doi: https://doi.org/10.1101/2025.10.09.680815; this version posted October 26, 2025. The copyright holder for this preprint (which was not certified by peer review) is the author/funder, who has granted bioRxiv a license to display the preprint in perpetuity. It is made available under aCC-BY 4.0 International license.

(D) Outline of the rapid capture of DMVs adopted from organelle IP[27-29] (top), as well as metabolic labeling for capturing the nascent mRNAs (bottom).

(E) Assessment of DMV IP. Shown are immunoblots of cytoplasmic inputs and GFP IPs of cells infected with MHV-A59 or MHV-Δ2-GFP3 probed for viral protein nsp9 and various cytoplasmic markers.

(F) Tail-length distributions of bulk and nascent mitochondrial mRNAs. Plotted are CDFs of poly(A)-tail lengths of total cytoplasmic and nascent mitochondrial mRNAs (input and 5-EU, respectively).

bioRxiv preprint doi: https://doi.org/10.1101/2025.10.09.680815; this version posted October 26, 2025. The copyright holder for this preprint (which was not certified by peer review) is the author/funder, who has granted bioRxiv a license to display the preprint in perpetuity. It is made available under aCC-BY 4.0 International license.

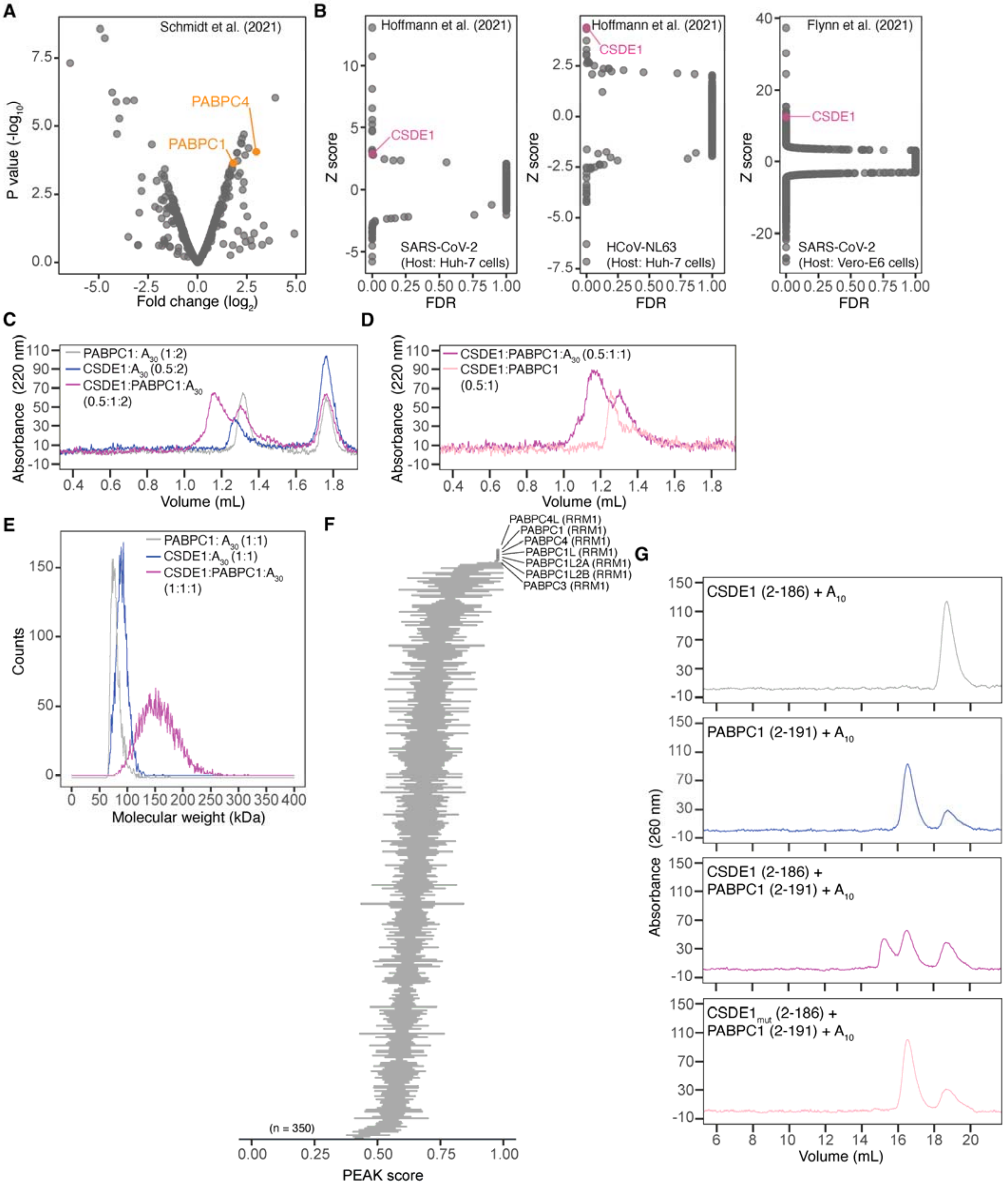

Figure S3. A host RBP forms a complex with viral poly(A) tail and PABPC1

(A) (A) Replot of RAP-MS data for SARS-CoV-2 mRNAs[36]. Plotted are changes in abundance of RBPs pulled down with probes targeting SARS-CoV-2 genomic mRNA compared to proteins pulled down with probes targeting RNA component of mitochondrial RNA processing endoribonuclease (RMRP).

(B) Identification of CSDE1 as a pro-viral factor. Plotted are the ranked score of CSDE1 and other proteins found in CRISPR screens designed to identify host dependencies of SARS-CoV-2 infection[48,49].

bioRxiv preprint doi: https://doi.org/10.1101/2025.10.09.680815; this version posted October 26, 2025. The copyright holder for this preprint (which was not certified by peer review) is the author/funder, who has granted bioRxiv a license to display the preprint in perpetuity. It is made available under aCC-BY 4.0 International license.

(C) Formation of a ternary complex involving full-length CSDE1, PABPC1, and the poly(A) tail. Shown are size-exclusion chromatograms of the full-length components of the CSDE1–PABPC1–oligo$(A)_{30}$ complex.

(D) Preferential binding of CSDE1 to poly(A)-bound PABPC1. Shown are size-exclusion chromatograms of CSDE1 and PABPC1 in the absence or presence of the oligo$(A)_{30}$.

(E) Stoichiometry of the ternary complex involving CSDE1, PABPC1, and oligo$(A)_{30}$. Plotted are mass photometry profiles of the full-length components of the CSDE1–PABPC1–oligo$(A)_{30}$ complex.

(F) Predicted binding of CSD2 to the RRM1 domain of PABPC paralogs. Plotted are PEAK scores for a ColabFold[50] screen of multimers of CSD2 of CSDE1 and all RRM domains in the human proteome.

(G) Biochemical support for the AF3 model of the complex between CSDE1 and RRM1-RRM2 domain of PABPC1. Shown are size-exclusion chromatograms for absorbance at 260 nm, which enables detection of the RNA, for the experiment shown in Figure 3G.

bioRxiv preprint doi: https://doi.org/10.1101/2025.10.09.680815; this version posted October 26, 2025. The copyright holder for this preprint (which was not certified by peer review) is the author/funder, who has granted bioRxiv a license to display the preprint in perpetuity. It is made available under aCC-BY 4.0 International license.

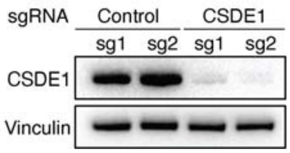

Figure S4. Efficiency of CSDE1 knockdown

Shown is an immunoblot probed for CSDE1 and Vinculin in control and CSDE1 KD NIH-3T3 cells targeted by one of two independent guide RNAs (sg1 and sg2).

bioRxiv preprint doi: https://doi.org/10.1101/2025.10.09.680815; this version posted October 26, 2025. The copyright holder for this preprint (which was not certified by peer review) is the author/funder, who has granted bioRxiv a license to display the preprint in perpetuity. It is made available under aCC-BY 4.0 International license.

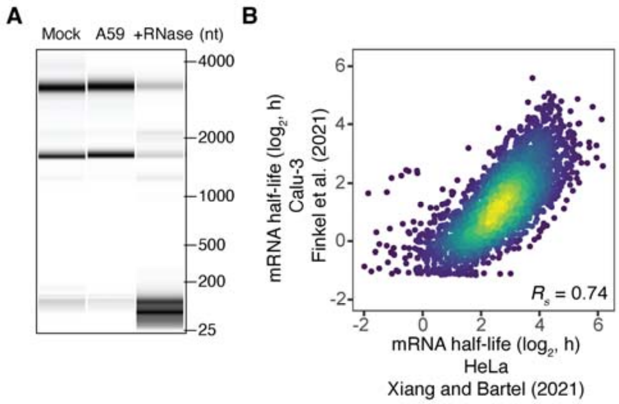

Figure S5. PABPC1 activity is limiting during infection, which destabilizes short-tailed host mRNAs

(A) Lack of evidence for activation of RNase L during infection with MHV-A59. Shown are bioanalyzer traces of total RNA collected from mock and MHV-infected L2 cell lysates (11 hpi) as well as from RNAse I treated lysates.

(B) The correlation between half-lives of mRNAs in uninfected Calu-3 cells[57] and in HeLa cells transfected with control siRNAs[11].

bioRxiv preprint doi: https://doi.org/10.1101/2025.10.09.680815; this version posted October 26, 2025. The copyright holder for this preprint (which was not certified by peer review) is the author/funder, who has granted bioRxiv a license to display the preprint in perpetuity. It is made available under aCC-BY 4.0 International license.

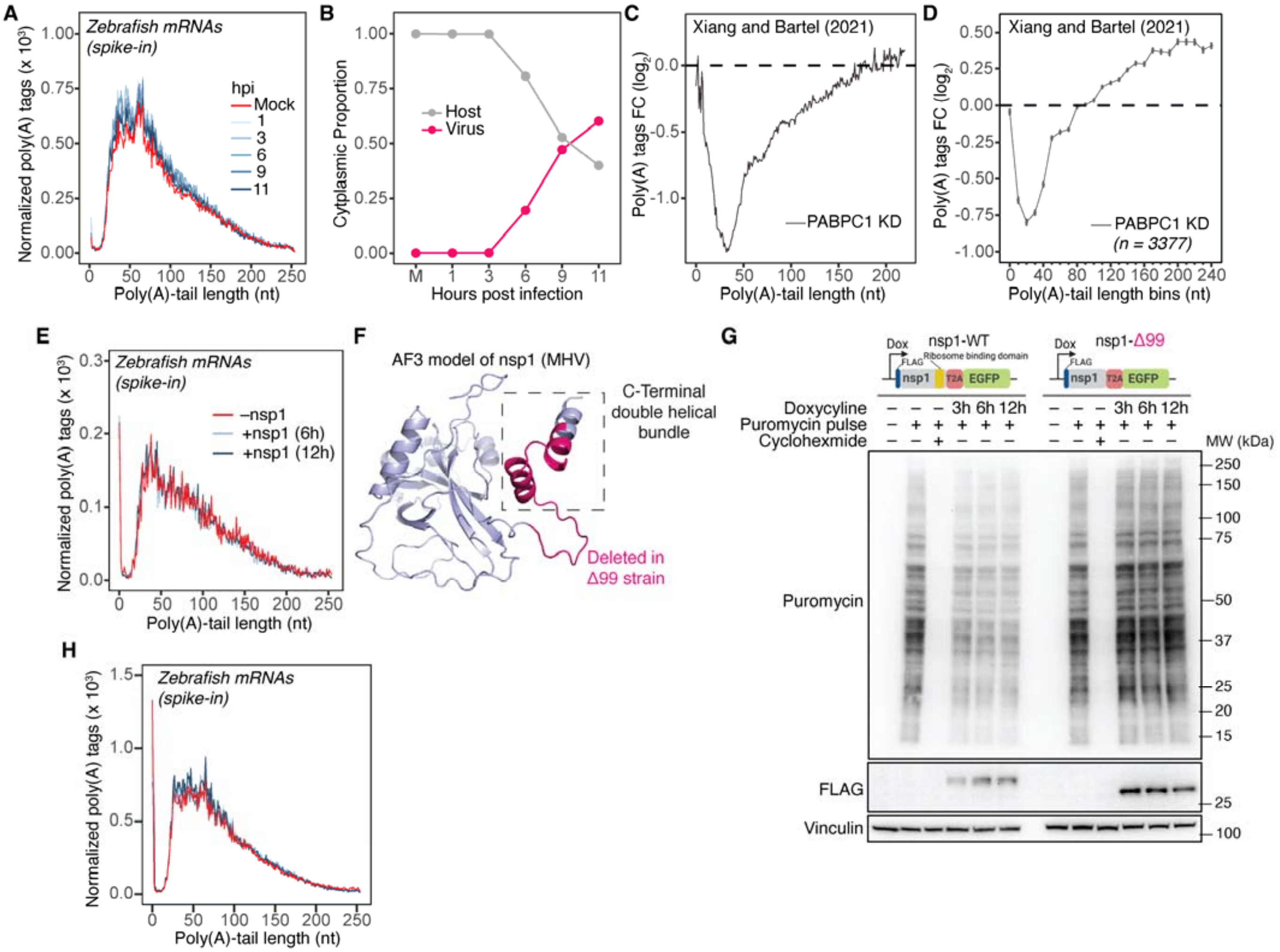

Figure S6. Host mRNAs with short poly(A) tails are preferentially degraded during infection

(A) Tail-length distributions of zebrafish mRNA poly(A) tags, used as spike-in controls for the experiment shown in Figure 6A.

(B) Domination of host cytoplasm by viral mRNAs. Plotted are the proportion of host and viral mRNAs in the total cytoplasmic mRNA population over the course of infection.

(C) Preferential degradation of short-tailed mRNAs upon PABPC1 knockdown in NIH-3T3 cells[11]. Otherwise, this panel is as in Figure 6B.

(D) Preferential degradation of short-tailed mRNAs upon PABPC1 knockdown in NIH-3T3 cells[11], analyzed after grouping mRNAs by gene; otherwise, as in Figure 6C.

(E) Tail-length distributions of zebrafish mRNA poly(A) tags, used as spike-in controls for the experiment shown in Figure 6D.

(F) AF3 predicted model of nsp1 from MHV. The putative ribosome-binding domain at the C terminus is indicated in a dashed box. The regions deleted in the MHV-Δ99 strain are shown in red.

(G) Impact of nsp1-Δ99 on global translation levels compared to nsp1; otherwise, as in Figure 5B.

(H) Tail-length distributions of zebrafish mRNA poly(A) tags, used as spike-in controls, for the experiment shown in Figure 6G.
